# *Saccharomyces boulardii* Improves Intestinal Epithelial Cell Restitution by Inhibiting αvβ5 Integrin Activation State

**DOI:** 10.1371/journal.pone.0045047

**Published:** 2012-09-20

**Authors:** Alexandra Canonici, Emilie Pellegrino, Carole Siret, Chloé Terciolo, Dorota Czerucka, Sonia Bastonero, Jacques Marvaldi, Dominique Lombardo, Véronique Rigot, Frédéric André

**Affiliations:** 1 Aix-Marseille Université, Centre de Recherche en Oncologie et Oncopharmacologie, Marseille, France; 2 Inserm UMR 9111, Marseille, France; 3 Team 4, Inflammation, Cancer, Cancer Stem Cells, INSERM U895, Centre Méditerranéen de Médecine Moléculaire, Nice, France; INRA, UR1282, France

## Abstract

Intestinal epithelial cell damage is frequently seen in the mucosal lesions of infectious or inflammatory bowel diseases such as ulcerative colitis or Crohn's disease. Complete remission of these diseases requires both the disappearance of inflammation and the repair of damaged epithelium. *Saccharomyces boulardii* (*Sb*, Biocodex) is a non-pathogenic yeast widely used as a preventive and therapeutic probiotic for the prevention and treatment of diarrhea and other gastrointestinal disorders. We recently showed that it enhances the repair of intestinal epithelium through activation of α2β1 integrin collagen receptors. In the present study, we demonstrated that α2β1 integrin is not the sole cell-extracellular matrix receptor involved during *Sb*-mediated intestinal restitution. Indeed, by using cell adhesion assays, we showed that *Sb* supernatant contains heat sensitive molecule(s), with a molecular weight higher than 9 kDa, which decreased αvβ5 integrin-mediated adhesion to vitronectin by competing with the integrin. Moreover, *Sb*-mediated changes in cell adhesion to vitronectin resulted in a reduction of the αvβ5signaling pathway. We used a monolayer wounding assay that mimics *in vivo* cell restitution to demonstrate that down-modulation of the αvβ5 integrin-vitronectin interaction is related to *Sb*-induced cell migration. We therefore postulated that *Sb* supernatant contains motogenic factors that enhance cell restitution through multiple pathways, including the dynamic fine regulation of αvβ5 integrin binding activity. This could be of major importance in diseases characterized by severe mucosal injury, such as inflammatory and infectious bowel diseases.

## Introduction

The colonic epithelium forms a continuous physical and functional barrier that protects the internal environment of the body from the fluctuating external milieu [1]. A variety of inflammatory gastrointestinal disorders, including infectious colitis and inflammatory bowel disease, result in the breakdown of the intestinal epithelial barrier and subsequent erosion and ulceration [1], [2], [3], [4]. The colonic epithelium possesses an innate ability to rapidly reseal superficial wounds, critical for the maintenance of barrier function and homeostasis. This process is dependent on the precise balance of migration, proliferation and differentiation of epithelial cells adjacent to the wounded area [1]. As with other epithelia of the gastrointestinal tract, the repair of damaged colonic mucosa initially requires cell restitution. This process is identified by stages of cell spreading and migration into the wound to restore epithelial continuity [1]. Restitution is followed by the proliferation and subsequent maturation and differentiation of the cells, allowing restoration of normal architecture and absorptive/secretory functions.

Colonic restitution has been found to be influenced by a broad spectrum of factors derived from the gastrointestinal environment, including host epithelial and lamina propria cells, resident microbiota, and both dietary and non-dietary components present in the gastrointestinal lumen [4], [5]. Both *in vitro* and *in vivo* studies have unveiled that adhesion-mediated signaling between cells and the extracellular matrix (ECM) is critical in the regulation of cell restitution [1], [6], [7], [8]. Moreover, *in vitro* studies have demonstrated that restitution is enhanced in the presence of ECM proteins [9], [10].

Interactions between cells and ECM are mainly mediated by cell surface adhesion molecules termed integrins. Integrins are glycosylated heterodimers composed of non covalently associated type I transmembrane α and β subunits [11]. In mammals, 18 α and 8 β subunits combine to form 24 distinct integrin receptors that bind various ECM ligands with different affinities [11]. Integrins allow a bi-directional flow of mechanochemical information across the plasma membrane and facilitate interactions between the ECM and the actin cytoskeleton. These integrin-mediated interactions are dynamically linked between either sides of the plasma membrane. The cytoskeleton controls the functional state of the integrins thus modulating their interaction with the ECM. Meanwhile integrin binding to the ECM changes the cell shape and the composition of the cytoskeleton beneath [11]. Integrin expression within the intestinal epithelium has been shown to vary, depending on their position along the crypt-villus-axis [12]. This suggests that these molecules are involved in epithelial cell migration. Moreover, during restitution, some integrins undergo a significant level of reorganization [13].

The probiotic yeast *Saccharomyces boulardii* (*Sb*) is widely used in a lyophilized form to treat and prevent antibiotic-associated and infectious diarrhea [14]. Recent *in vitro* and *in vivo* studies indicate that this probiotic interacts with pathogenic micro-organisms and resident microflora, as well as intestinal mucosa [15], [16], [17]. In addition, clinical trials have suggested that *Sb* can be effective in the treatment of inflammatory bowel diseases (IBD) [18], [19], [20] via modulation of host cell signaling pathways implicated in the pro-inflammatory response [3], [21], [22]. Furthermore, we have recently shown both *in vitro* and *in vivo* that *Sb* secretes factors that modulate intestinal epithelial cell restitution. This is in part through the activation of the α2β1 integrin collagen receptor signaling pathway [15].

However α2β1 integrin is not the sole cell-ECM receptor involved in colonic restitution, since α3β1 integrin/laminin and αvβ5 integrin/vitronectin (Vn) interactions are also known to regulate colonic restitution [23], [24]. This led us to determine whether *Sb* supernatant (*Sb*S) has an impact on cell migration via a α2β1 integrin-independent mechanism. In the present study, we have shown that *Sb*S contained one or more heat-sensitive molecules which blocked the αvβ5-mediated cell-Vn interaction. This change in cell-ECM adhesion could be responsible for the observed increase in cell restitution.

## Methods

### Cell culture

The human colonic adenocarcinoma cell line HCT-8/E11 (gift of Pr M. Bracke-Ghent, Belgium) was routinely cultured as previously described [25]. Cells were cultured on plastic dishes until they reached confluency. These cellular monolayers consisted of polarized cells joined by tight junctions, which exhibited well developed apical microvilli, allowing the study of processes involved in intestinal epithelial cell physiology. The human colonic adenocarcinoma cell line HT29-D4, established in our laboratory, was cultured as previously described [15].

### Yeast culture supernatant

Lyophilized *Sb* was provided by Biocodex laboratories (Gentilly, France). *Sb*S was prepared as described previously [15], [26]. In brief, *Sb* (100 mg/ml) was rehydrated in epithelial cell culture media RPMI 1640 without fetal calf serum and incubated overnight at 37°C in aerobiosis condition. Conditioned media were centrifuged at 20,000× *g* for 15 min to separate the yeast cells from the supernatant and the supernatant collected. The supernatant was passed through 0.22 µm filters (Fisher Scientific) to remove cell debris. Serial dilutions ranging from 1/8 to 1/128 were performed in RPMI. None of the diluted supernatants affected cell viability, as verified by the trypan blue exclusion test. In some experiments, *Sb*S was fractionated through a Pierce concentrator 9 kDa MWCO filter (Thermo Scientific). To confirm that the filtration discriminates between molecules superior and inferior to 9 kDa, fractions were analyzed by SDS PAGE. We never detected >9 kDa molecules in the <9 kDa fraction (not shown).

### Cell adhesion assay

Adhesion substrata were prepared by coating flat-bottom 96-well microtiter plates overnight at 4°C with 50 µl of vitronectin (Vn) or fibronectin (Fn) diluted in phosphate-buffered saline (PBS) at the indicated concentrations. Coated wells were blocked with 1% BSA in PBS for 30 min, and then washed twice with PBS. HCT-8/E11 single-cell suspensions (50,000 cells/0.1 ml) prepared in DMEM containing 0.2% BSA (adhesion buffer) were seeded in substratum-coated wells and allowed to adhere for 2 h at 37°C. Unattached cells were removed by 4 gentle washes with adhesion buffer and residual attached cells were fixed by 1% glutaraldehyde. After staining with 0.1% crystal violet, cells were lyzed with 1% SDS and the optical density was measured at 600 nm by a microplate reader. When required, 10 µg/ml of function-blocking anti-integrin mAbs (rat anti- αv subunit, clone 69.6.5; Beckman Coulter or mouse anti -α5 subunit, clone SAM-1; Millipore) were added during the time of cell adhesion.

### Cell spreading assay

24-well plates were coated at 4°C overnight with 250 µl of either Vn or Fn at 3.15 µg/ml. Coated wells were blocked with 0.5% BSA in PBS for 30 min and then washed twice with PBS. HCT-8/E11 single-cell suspensions (25,000 cells/0.5 ml) were seeded in substratum-coated wells and allowed to adhere for 2 h at 37°C. Cells were not washed in order to preserve both attached and unattached cells. The ratio of cells that have filopodia and/or lamellipodia over total cells was calculated by counting cells microscopically and was referred to the percentage of cell spreading. Cell spreading was determined by counting cells in 5 microscopic fields per well.

### Flow cytometry analysis

HCT-8/E11 cell surface expression of integrins was determined by flow cytometry as previously described [27]. Briefly, sub-confluent cells were harvested and resuspended in DMEM containing 20% fetal calf serum and 1% BSA. The single cell suspension (10^6^ cells/ml) was incubated for 1 h at 4°C in the presence of 10 µg/ml anti-integrin mAbs (rat anti -α6 subunit: clone GOH3, Beckman Coulter; rat anti-αv subunit: clone 69-6-5, Beckman Coulter; mouse anti-β1 integrin: clone K20, Beckman Coulter; mouse anti αvβ5 clone P1F6, Millipore). Cells were rinsed once with ice-cold DMEM containing 0.1% BSA and then incubated for 45 min at 4°C with the appropriate Alexa Fluor 488-conjugated antibody. After washing, cells were fixed with 2% formaldehyde and cell-bound fluorescence was quantified using a Becton-Dickinson FACScan flow cytometer. Non-specific labeling was determined by incubating cells with the secondary Alexa 488-conjugated Ab alone.

### Focal adhesion labeling

HCT-8/E11 cell suspensions (50,000 cells/0.5 ml) were either treated with or without *Sb*S and seeded in Vn or Fn-coated wells (3.15 µg/ml). They were allowed to adhere for 2 h at 37°C. Cells were fixed for 20 min at 37°C with 3% formaldehyde in PBS, permeabilized by incubation in PBS/0.1% saponin for 30 min, then incubated for 1 h in PBS containing 4% BSA (w/v). αv integrin subunit, vinculin and tyrosine phosphorylated proteins were detected with 10 µg/ml AMF-7 (Beckman-Coulter), hVIN-1 (Sigma), or PY20 (Millipore) Abs, respectively. After 4 washes in PBS, cells were incubated with Alexa Fluor 488-conjugated sheep Ig (20 µg/ml), raised against mouse and rabbit Igs, for 1 h, then washed and mounted in ProLong Gold® (Invitrogen). Images were captured and analyzed using a SP5 Leica confocal microscope equipped with LAS AF Lite software. Focal adhesion (FA) labeling with anti-vinculin, -αvβ5 and -phosphotyrosine Abs was quantified using ImageJ software by measuring the area of each focal adhesion for all cells. Approximately 40 cells for each condition were analyzed.

### Immunohistochemistry

Groups of 6-week old C57BL6J female mice (n = 4) were force-fed daily with 200 µL RPMI 1640 or with 200 µL *Sb*S, for 1 week. After the sacrifice of mice, segments of intestine were frozen in liquid nitrogen and cryosectioning (section thickness: 8 µm) was performed. Samples were fixed in acetone (−20°C for 10 min), rehydrated then blocked for 30 min in PBS containing 4% (w/v) BSA. αvβ5 integrin and E-cadherin were detected by an overnight incubation at 4°C with a rabbit anti-β5 integrin subunit (clone H-96; Santa Cruz Biotechnology) and a rat anti-E-cadherin mAb (clone ECCD-2, Millipore), respectively. After 3 washes in PBS, cells were incubated with Alexa Fluor 594- or 488-conjugated sheep Ig (20 µg/ml), raised against rabbit and rat Igs respectively, for 1 h, then washed and mounted in ProLong Gold® (Invitrogen). Non-specific labeling was determined by incubating cells with a mixture of rabbit Igs and rat Igs followed by an incubation with the secondary Alexa 594-and 488-conjugated Abs. Images were captured and analyzed using a SP5 Leica confocal microscope equipped with LAS AF Lite software.

All animal experiments were performed in accordance with the regulations of our institution's ethics commission. They were conducted following the APS Guiding Principles in the Care and Use of Animals. The study was approved by the Ethics Committee in Animal Experimentation of Centre Méditerranéen de Médecine Moléculaire (C3M), Nice, France (protocol 3/2010).

### Cell migration assay

Monolayers of differentiated HCT-8/E11 cells were wounded using a sterile tooth-pick and incubated with or without various dilutions of *Sb*S. Plates were placed in a temperature and CO_2_-controlled chamber mounted on a Nikon TE2000 inverted microscope. Images were captured every 5 min for a total observation period of 5 h using a Cool SnapHQ camera (Princeton Instruments) through a 10× objective lens. For each wound, 10 measurements of wound width were recorded. To assess the role of the integrins in *Sb*S enhanced enterocyte migration, 10 µg/ml of the following function-blocking anti-integrin mAbs were used: αv integrin (69.6.5, Beckman Coulter), αvβ3integrin (mouse IgG1, LM 609 clone, Millipore), αvβ5integrin (mouse IgG1, P1F6 clone, Millipore), or αvβ6 (mouse IgG2a, 10D5 clone, Millipore). Rat-anti dipeptidyl peptidase IV (clone 5H2, gift of S. Maroux, CNRS, Marseille, [28]), and mouse anti-α5 subunit (mouse IgG2, clone SAM-1; Millipore) were used as isotypic Abs controls. Abs were added both 1 h before wounding and during the period of cell migration.

### Detection of tyrosine-phosphorylated FAK

HCT-8/E11 cell suspensions (800,000 cells/1 ml), prepared in adhesion buffer, were seeded onto Vn- or Fn-coated wells and allowed to adhere for 1 h. Both adherent cells and cells in suspension were lyzed as previously published [29]. Equal amounts of cell lysates (25 µg) were resolved by SDS-PAGE and blotted onto a nitrocellulose sheet. Membranes were blocked with PBS containing 4% BSA and probed overnight at 4°C with a mouse anti-Y397-FAK (Invitrogen). Blots were then revealed by chemiluminescence after incubation with the appropriate horseradish peroxidase-conjugated secondary Ab (Amersham). Loading amounts were verified by probing the blot with a rabbit-anti FAK (Ozyme) Ab or with a rabbit-anti-β tubulin (Sigma) Ab.

### Knock-down of αv integrin by small interfering RNA

αv integrin suppression in HCT-8/E11 cells was performed as previously described [30].

### Statistical analysis

Unless noted, data are presented as the means ± S.D. for three independent experiments performed in triplicate. Comparison between the two conditions was made by using the Mann–Whitney test for sampling<30. *P*<0.05 was considered statistically significant in all analyses and is indicated by ‘***’ when <0.001, ‘**’ when <0.01 and ‘*’ when <0.05.

## Results

### αvβ5 integrin supports *Sb*S-mediated cell migration

**Figure 1:**
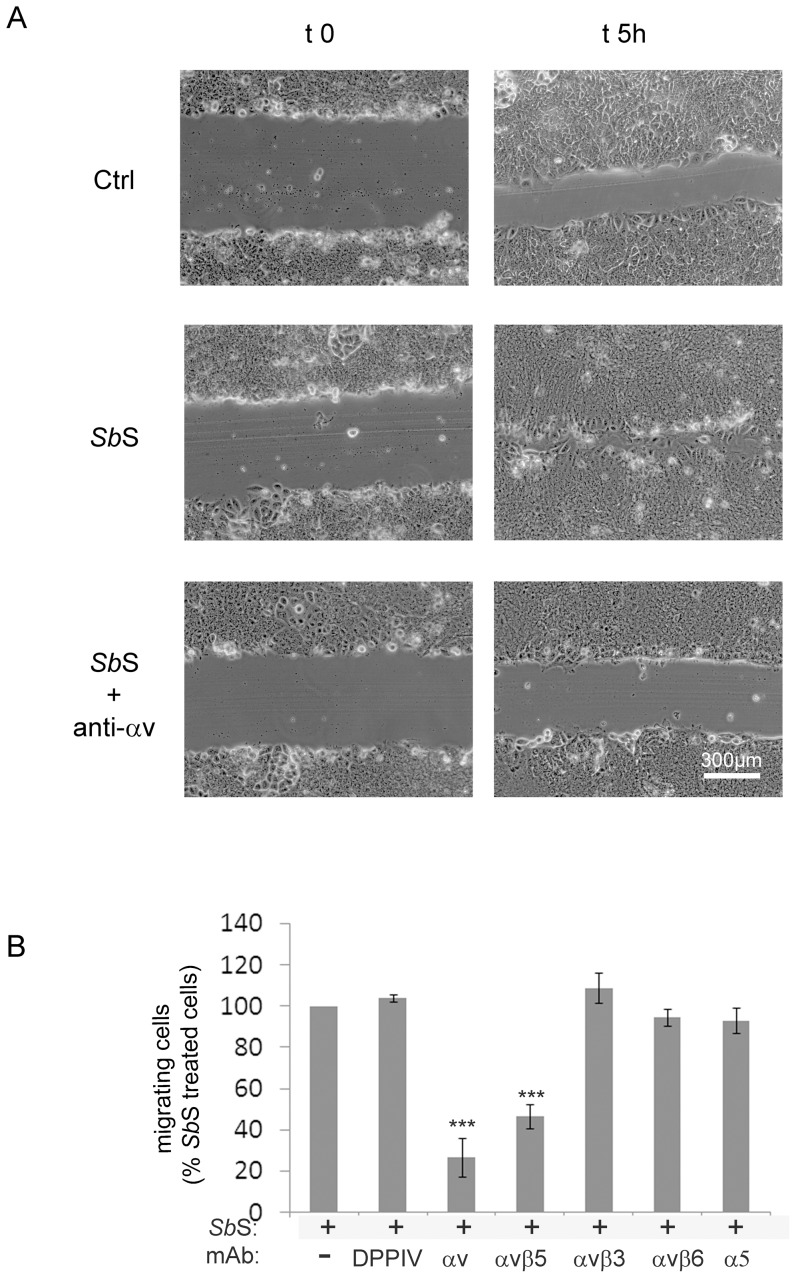
αvβ5 integrin is required for *Sb*S-stimulated cell restitution. (A) Polarized HCT-8/E11 cell monolayers were pretreated without or with 10 µg/ml of an anti- αv integrin mAb (anti-αv) for 1 h. Cell monolayers were then wounded as described in Methods and incubated for 5 h in the absence (ctrl) or presence of *Sb*S in a medium either containing anti-αv integrin mAb or not. Phase contrast images were acquired at the indicated times. Data shown are from a representative experiment out of 3 performed. Scale bar: 300 µm. (B) HCT-8/E11 cell monolayers were wounded and incubated with *Sb*S. The monolayers were further incubated without (-) or with the following function-blocking anti-mAbs 1 h before wounding and during cell migration: αvintegrin (αv), αvβ3 integrin (αvβ3), αvβ5 integrin (αvβ5), or αvβ6 integrin (αvβ6). Wound closure was determined as described in Methods. Results are expressed as the percentage of cell migration compared to *Sb*S-treated cells without mAbs. Data represent the mean+SD of 5 separate experiments. Rat-anti DPP IV (DPPIV) and mouse anti-α5 subunit (α5) were used as isotypic Abs controls. *** P<0.001.

We previously reported both *in vitro* and *in vivo* that *Sb*S improves intestinal cell restitution through activation of the α2β1 integrin collagen receptor [15]. However it is clear that α2β1 integrin is not the sole receptor involved in this process since a function-blocking anti-αv subunit integrin mAb partially inhibited *Sb*S-mediated cell migration (figure **1** and supplemental video **S1** and **S2**). Quantification of the surface area recovered by the cells indicated that the anti- αv subunit integrin mAb inhibited the migration capacity of *Sb*S-treated HCT-8/E11 cells by approximately 70% (figure **1**). To delineate which αvβ integrin was involved in the migratory process we incubated HCT-8/E11 cells with function-blocking anti-αvβ3, anti-αvβ5or anti-αvβ6integrin mAbs during cell migration. As observed in figure **1B**, only the anti-αvβ5integrin mAb partially blocked both control and *Sb*S-induced HCT-8/E11 cell migration.

### αvβ5 integrin is redistributed in *Sb*S force-fed mice

**Figure 2:**
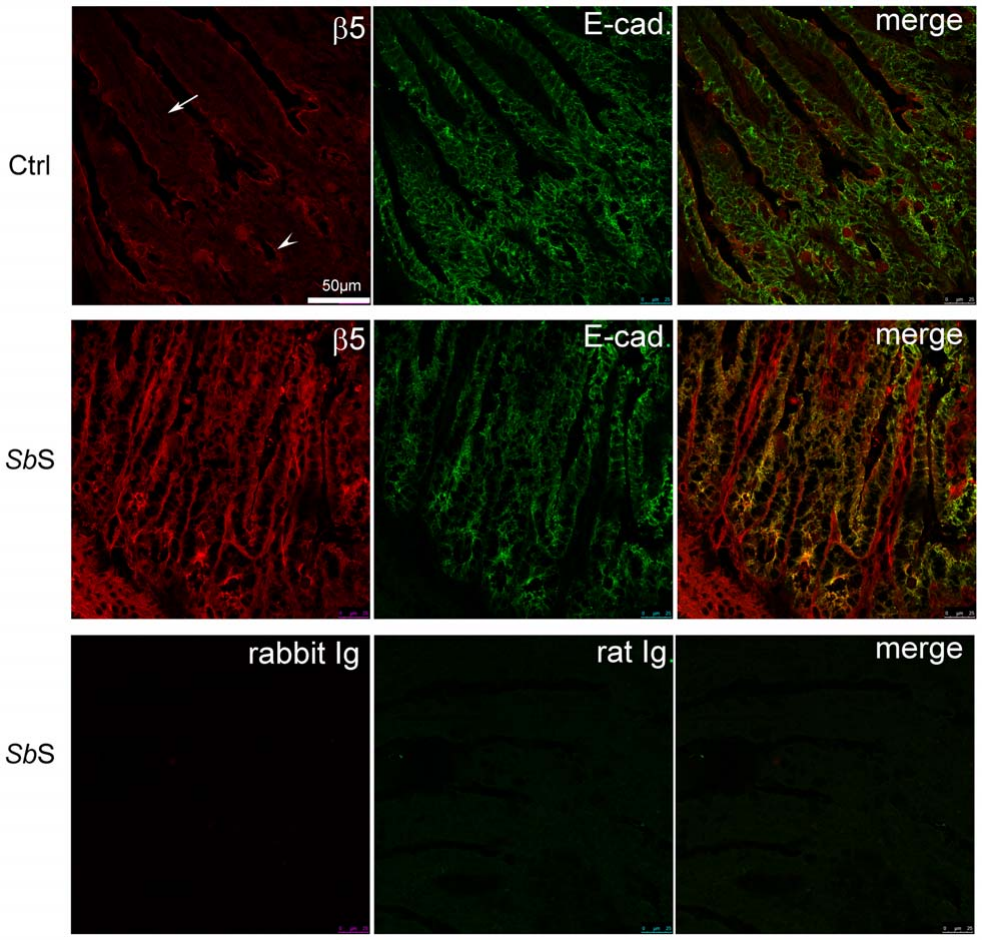
αvβ5 integrin is relocalized in the intestine of *SbS* force-fed mice. Mice were daily force-fed for one week with unused culture medium for *Sb* (Ctrl) or *Sb*S (*Sb*S). Frozen sections of small intestinal tissues were co-stained with a rabbit anti-β5-β integrin subunit (β5) and a rat anti E-cadherin (E-cad). Sections were then incubated with a mixture of Alexa 594-conjugated and Alexa 488-conjugated secondary antibodies against rabbit and mouse IgG, respectively. Rabbit Ig and rat Ig correspond to isotype controls Abs. Colocalized pixels appear in yellow in merged images. Each image is a representative image taken from tissue sections of four mice. The arrow and the arrowhead point out a villus and a crypt, respectively.

Since αvβ5 integrin is involved in *Sb*S-induced cell migration *in vitro*, we next evaluated whether *Sb*S affected the distribution of αvβ5 integrin *in vivo*. C57BL6J mice were force-fed daily with either RPMI 1640 or *Sb*S, for 1 week. We verified that *Sb*S treatment did not alter the mucosal architecture. The distribution of β5 integrin subunit was determined by immunohistochemistry on intestinal tissues (figure **2**). In control mice, β5 subunit was abundantly detected at the surface of cells located in the crypts and along the apical membrane domain of cell present in the villus. By contrast, in *Sb*S force-fed mice, β5 positive cells were mainly redistributed to the basal surfaces of enterocytes in the villus (figure 2B). The pattern of E-cadherin expression was also analyzed along the crypt-villus axis in order to locate epithelial cells. As observed on figure **2**, E-cadherin positive cells also expressed β5 integrin subunit. Moreover, upon *Sb*S treatment, β5 integrin was re-distributed at the basal side of the epithelial intestinal cells. This suggests that αvβ5integrin participates in *Sb*S-mediated epithelial cell migration. However, it should be noted that *Sb*S increased the β5 subunit integrin expression level of E-cadherin negative cells located into the lamina propria, suggesting that *Sb*S could also act on non-epithelial cells, as recently described [31].

### *Sb*S modulates αv integrin-mediated adhesion

**Figure 3:**
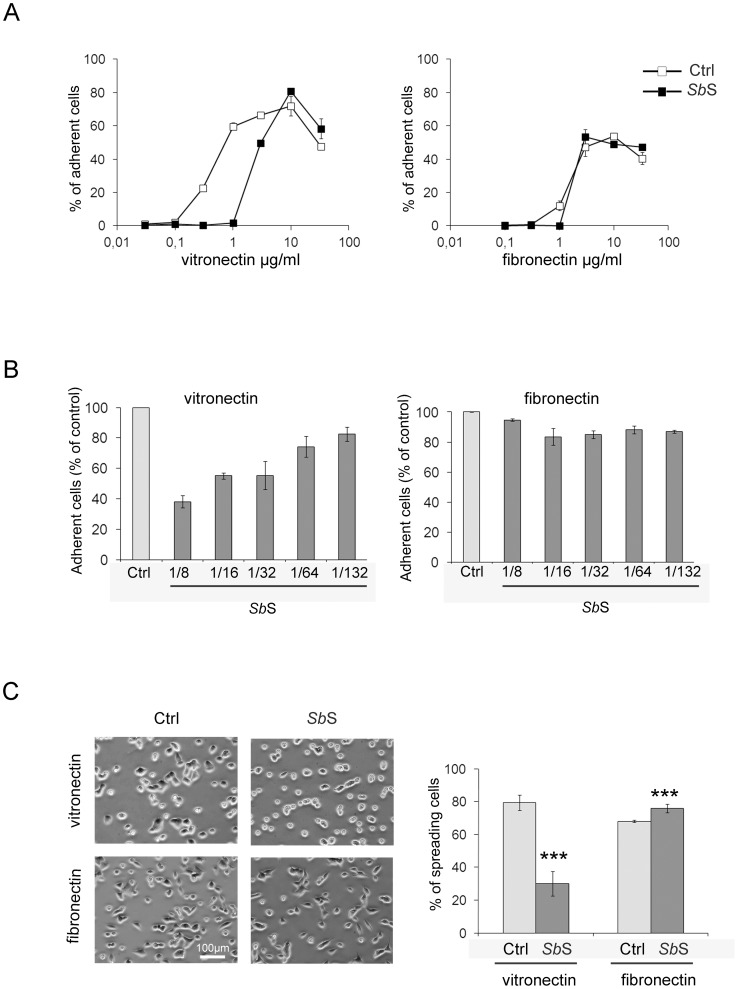
*Sb*S down-modulates cell adhesion to vitronectin. (A) Isolated HCT-8/E11 cells were treated with or without *Sb*S (dilution 1/8) and plated on either fibronectin or vitronectin at the indicated concentrations. Cell-ECM adhesion was evaluated as described in Methods. Results are expressed as the percentage of cell adhesion. Data represent the mean+SD of 3 separate experiments. (B) HCT-8/E11 cells were treated with or without dilutions of *Sb*S and plated on vitronectin (Vn). Cell-vitronectin adhesion was evaluated as described in Methods. (C) HCT-8/E11 cells were treated with or without *Sb*S, seeded in vitronectin-coated or fibronectin-coated wells and allowed to adhere for 2 h at 37°C. Spreading cells were counted microscopically. Results are expressed as the percentage of spreading cells. Data represent the mean+SD of 3 separate experiments. *** P<0.001.

As regulation of cell adhesion and cell spreading are essential for cell migration, we next examined whether *Sb*S altered HCT-8/E11 cell attachment and spreading to both Fn and Vn. Vn is a ligand of αvβ5 integrin whereas Fn is recognized by α5β1 and αvβ3 integrins but not by αvβ5 integrin. However the primary sequence motif for the ligand binding of these integrins is a tripeptide RGD sequence [32]. Cells were either treated with *Sb*S or not, then allowed to attach to increasing amounts of purified Fn or Vn. HCT-8/E11 cells attached to the two matrices tested (figure **3A**). *Sb*S did not alter cell adhesion to Fn. However, the percentage of cells adherent to Vn was dramatically decreased by *Sb*S. As depicted by figure **3B**, *Sb*S down-modulated cell attachment to Vn in a dose-dependent manner, with a maximal effect obtained with a 1/8 dilution of *Sb*S. Since cell spreading is initiated immediately after cell contact with matrix proteins, we also determined whether *Sb*S affected cell spreading on both Fn and Vn. As observed in cell adhesion assays, *Sb*S dramatically down-modulated the capacity of cells to spread on Vn. On the other hand, it weakly, albeit significantly, increased HCT-8/E11 cell spreading on Fn (Figure **3C**).

**Figure 4:**
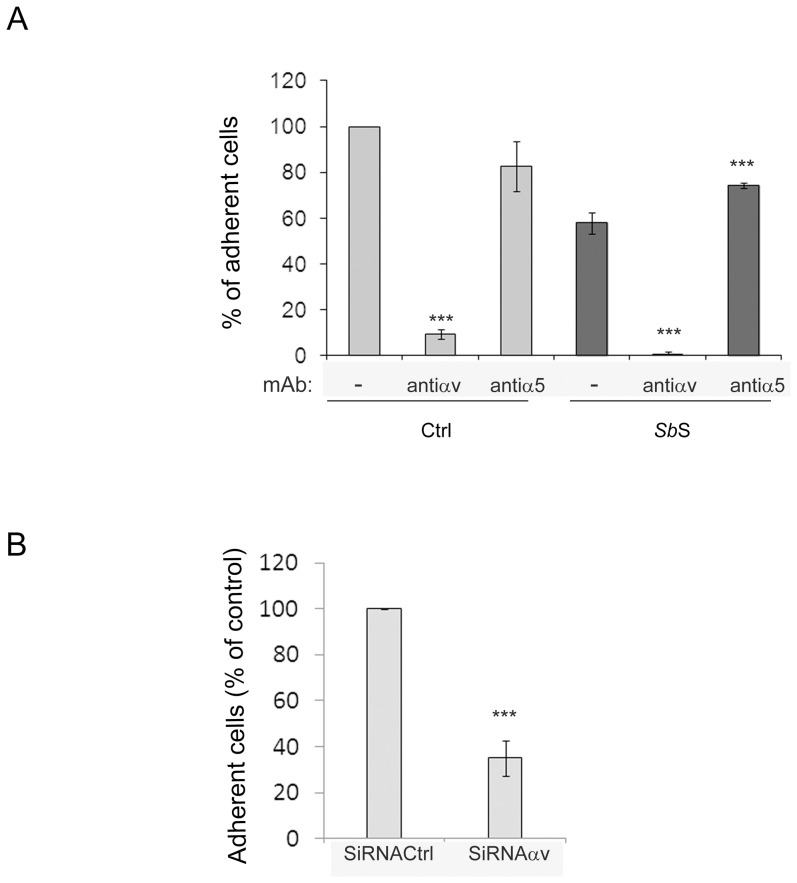
αvβ5 integrin is required for HCT-8/E11 cell interaction with vitronectin. (A) Isolated HCT-8/E11 cells were incubated without (Ctrl) or with *Sb*S (*Sb*S) in the absence (-) or presence of either anti-αv or -α5 integrin mAbs. Cells were then seeded on vitronectin (3,15 µg/ml). Cell adhesion was evaluated as described in Methods. Results are expressed as the percentage of cell adhesion compared to untreated cells (Ctrl). Data represent the mean+SD of 3 separate experiments. (B) HCT-8/E11 cells were transfected with anti -αv-integrin siRNAs (SiRNAαv) or scramble oligos (SiRNACtrl) for 48 h. Cells were then plated on vitronectin for 2 h. Cell-ECM adhesion was evaluated as described in Methods. Results are expressed as the percentage of cell adhesion. Data represent the mean+SD of 3 separate experiments. ***P<0.001.

To delineate which α and β integrin subunits were affected by *Sb*S during both cell adhesion and spreading to Vn, we performed adhesion assays in the presence of anti-integrin function-blocking mAbs. The interaction between HCT-8/E11 cells and Vn was mediated solely by the αv integrin subunit (Figure **4A**) whatever the condition tested. An anti- α5integrin subunit that blocked the HCT-8/E11 cell-Fn interaction (not shown) did not affect cell adhesion to Vn. It should be noted that when HCT-8/E11 cells were treated with *Sb*S, blockade of the α5 integrin subunit led to a weak increase in cell adhesion to Vn (Figure **4A**). This may suggest a functional interplay between αv and α5 integrin subunits. As observed in Figure **4B**, αv subunit silencing also dramatically blocked cell adhesion to Vn, confirming that the αv integrin subunit is the α subunit receptor for Vn in HCT-8/E11 cells.

### *Sb*S competes with αv integrin for binding to vitronectin

**Figure 5:**
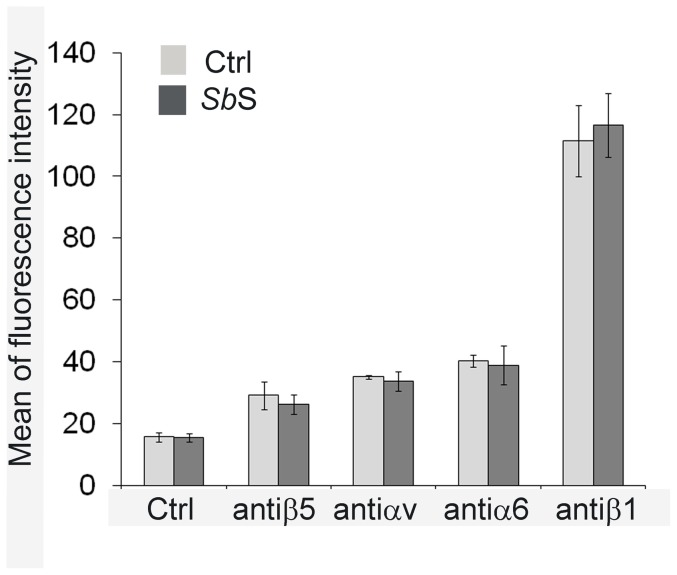
Impact of *Sb*S on integrin expression. Sub-confluent HCT-8/E11 cells were incubated for 5 h with (dark bars) or without (light bars) *Sb*S, then harvested and resuspended in the presence of anti-integrin subunit mAbs. After incubation with the appropriate secondary Alexa 488-conjugated Ab, cell-bound fluorescence was quantified using a Becton-Dickinson FACScan flow cytometer. Non-specific labeling was determined by incubating cells with the secondary Alexa 488-conjugated Ab alone (Ctrl). Data represent the mean+SD of 6 separate experiments.

Modulation of integrin expression at the cell surface may alter cell migration. To determine whether *Sb*S modified integrin expression, we quantified the integrin subunits located at the plasma membrane by flow cytometry analysis. As shown in figure **5**, *Sb*S did not significantly alter expression levels of the αv, β5, α6 and β1 integrin subunits.

**Figure 6:**
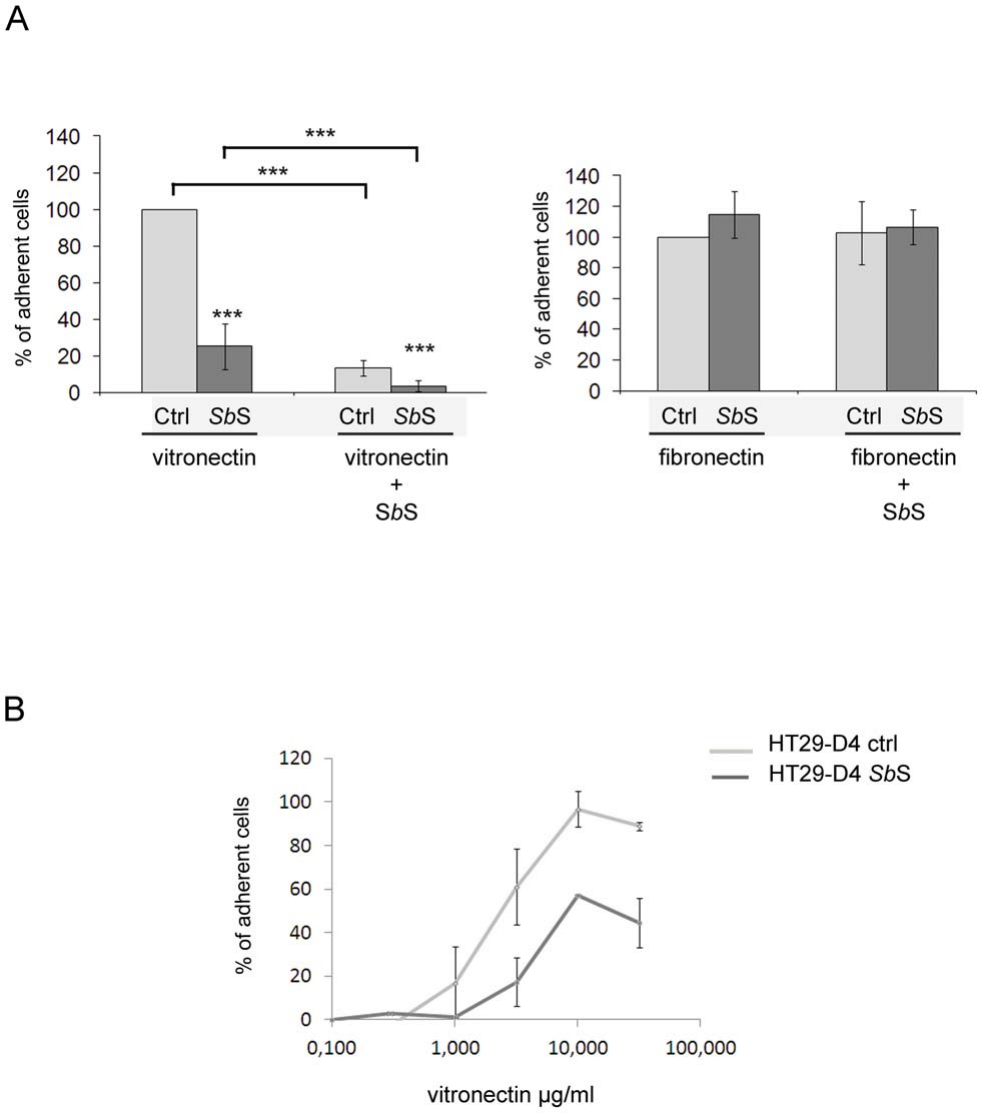
*Sb*S blocked αvβ5 integrin interaction with vitronectin. (A) Vitronectin- or Fibronectin-coated plates were incubated in the absence (Vitronectin and Fibronectin) or presence of *Sb*S (vitronectin+ *Sb*S and fibronectin+ *Sb*S) prior to addition of cells. Isolated HCT-8/E11 cells incubated without (light bars) or with *Sb*S (dark bars) were then seeded on plates. Cell adhesion to ECM was evaluated as described in Methods. Results are expressed as the percentage of cell adhesion compared to untreated cells (Ctrl). Data represent the mean + SD of 3 separate experiments. (B) Isolated HT29-D4 cells were treated with or without *Sb*S (dilution 1/8) and plated on vitronectin at the indicated concentrations. Cell-vitronectin adhesion was evaluated as described in Methods. Results are expressed as the percentage of cell adhesion. Data represent the mean+SD of 3 separate experiments. *** P<0.001.

Since *Sb*S inhibited cell adhesion to Vn without modulation of αv integrin subunit expression, we postulated that it blocked αv integrin-Vn interaction. To confirm this hypothesis, ECM-coated plates were incubated in the presence of *Sb*S prior to addition of cells. We first determined that cells did not adhere to *Sb*S coated plates (not shown). Addition of *Sb*S to Vn-coated plates impaired cell adhesion (Figure **6A**). Moreover, the same results were obtained when *Sb*S-coated plates were incubated with Vn prior to cell adhesion (not shown). However, when the same experiments were performed using Fn instead of Vn, no change in cell attachment was found (Figure **6A**). This suggests that *Sb*S specifically interacts with Vn and blocks αv-dependent HCT-8/E11 adhesion. Furthermore, similar results were observed using another intestinal cell line HT29-D4 (Figure **6B**), indicating that the observed phenomenon could be common to intestinal cell lines.

### *Sb*S regulates the αvβ5 integrin signaling pathway

**Figure 7:**
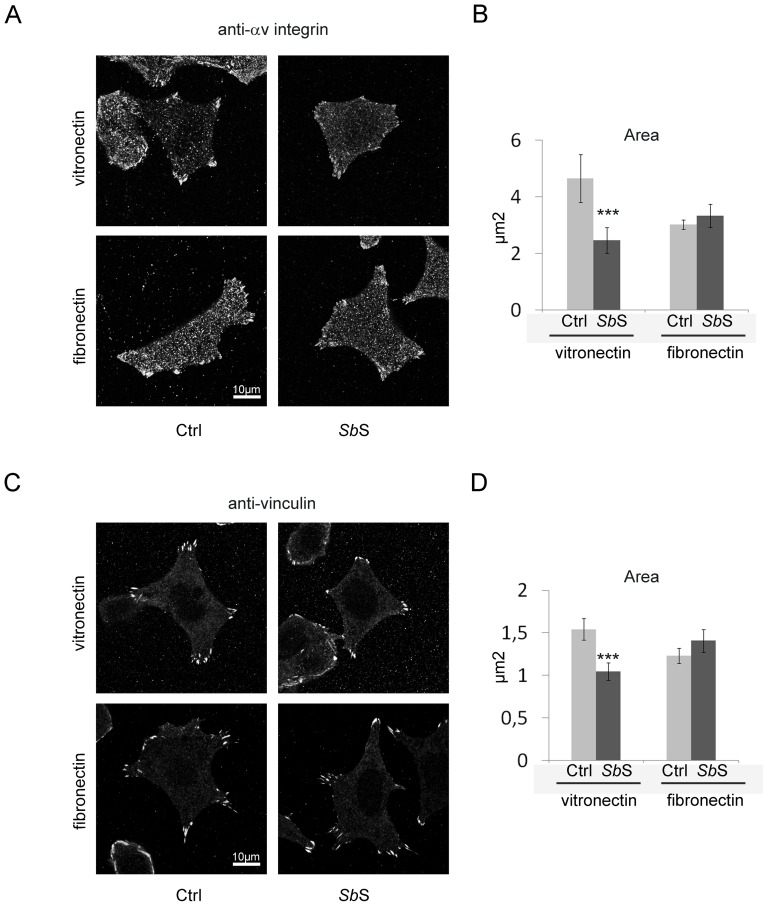
*Sb*S alters the organization of adherence structures when cells are plated on vitronectin. HCT-8/E11 cells were plated on either fibronectin or vitronectin for 2 h and then fixed. αv integrin (A) or vinculin (C) were detected using specific Abs. Focal adhesion labeling was quantified by measuring the area of each focal adhesion for all cells (B and D). Data represent the mean+SD of 3 separate experiments. *** P<0.001.

Activation of αvβ5 integrin leads to remodeling of the actin cytoskeleton through the formation and activation of a large signaling complex called focal adhesion. This adhesion complex contains enzymes and scaffolding molecules including FAK and vinculin [32]. Since *Sb*S modulated HCT-8/E11 adhesion to Vn, we first checked whether *Sb*S regulated αvβ5 integrin activation. We first analyzed the impact of *Sb*S on the organization of adherence structures after plating cells on Vn. As depicted by Figure **7A**, αv integrin subunit staining revealed that control cells plated on Vn, exhibited focal adhesion structures. However, αv integrin subunit staining in *Sb*S treated cells displayed less focal adhesion structures. Analysis of the αv integrin subunit detected at the cell-substratum interface revealed that *Sb*S markedly decreased the area of focal adhesion sites (Figure **7B**). The same results were obtained with vinculin (Figures **7C** and **7D**). These results indicate that treatment with *Sb*S is associated with structural modifications of focal adhesion structures. Interestingly this effect was not observed when Fn was used as a substrate (Figures **7A–B** and **7C–D**).

**Figure 8:**
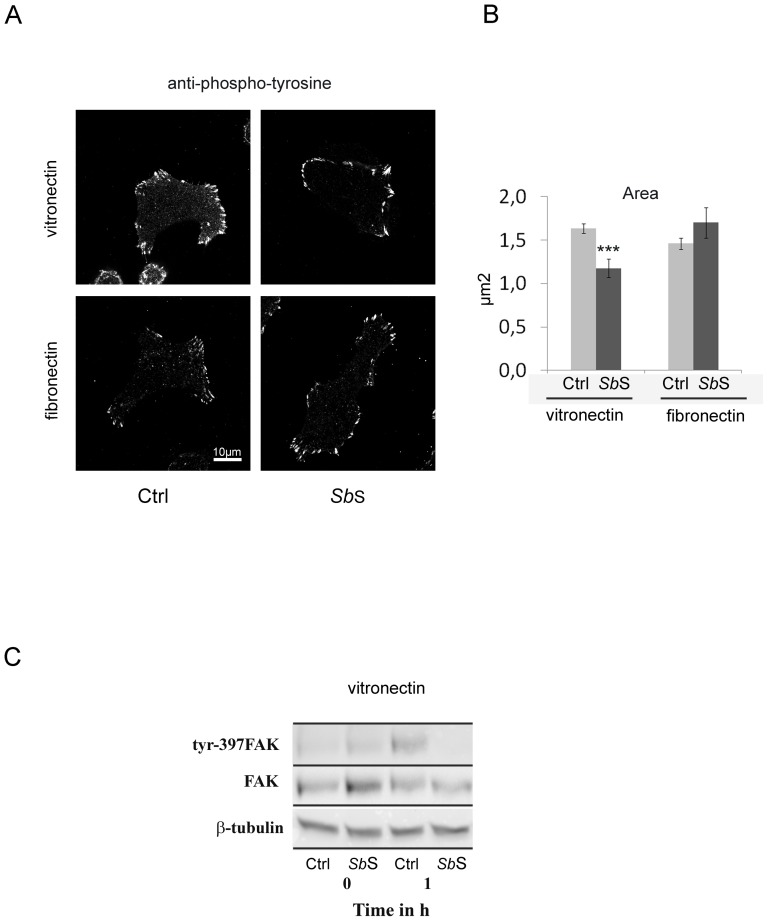
*Sb*S alters the functionality of adherence structures. (A) HCT-8/E11 cells were plated on either fibronectin or vitronectin for 2 h and then fixed. Tyrosine phosphorylated proteins were stained using a PY20 mAb. (B) Focal adhesion labeling was quantified by measuring the area of each focal adhesion for all cells. Data represent the mean+SD of 3 separate experiments. (C) HCT-8/E11 cells were allowed to adhere on vitronectin after *Sb*S pretreatment. The phosphorylation of tyrosine residues in FAK (Tyr-397FAK) was determined after cell lysis at the indicated times of cell adhesion. Samples were analyzed by western blot analysis. Equal amounts of protein were analyzed and loading amounts were verified by probing the blot with anti-FAK (FAK) or anti- β-tubulin (β-tubulin) Abs. *** P<0.001.

We next analyzed whether *Sb*S the αvβ5-mediated signaling pathway. We first determined whether *SbS* affected protein tyrosine phosphorylation by immunolocalization. In untreated cells, the mAb PY20 directed against the phosphorylated tyrosine residue, mainly stained cell-ECM contact sites, namely focal adhesion structures (Figure **8A**). However, *Sb*S treatment promoted a decrease in the area of the tyrosine phosphorylated focal adhesion sites (Figures **8A** and **8B**). This suggests that *Sb*S may negatively modulate the activity of focal adhesion structures. To confirm the impact of *Sb*S on the αvβ5 signaling pathway, cells were either treated with *Sb*S or not and plated on a Vn-coated surface for 1 h. The tyrosine phosphorylation status of FAK post-adhesion was determined by Western blot using an anti-Y397 FAK mAb. As depicted in figure **8C**, adhesion of control cells to Vn promoted FAK tyrosine phosphorylation on residue 397. Interestingly, *Sb*S decreased the tyrosine phosphorylation level of FAK, confirming that *Sb*S may regulate αvβ5 integrin activation (Figure **8C**).

### *Sb*S contains a heat-sensitive molecule, >9 kDa, that regulates both cell adhesion and migration

**Figure 9:**
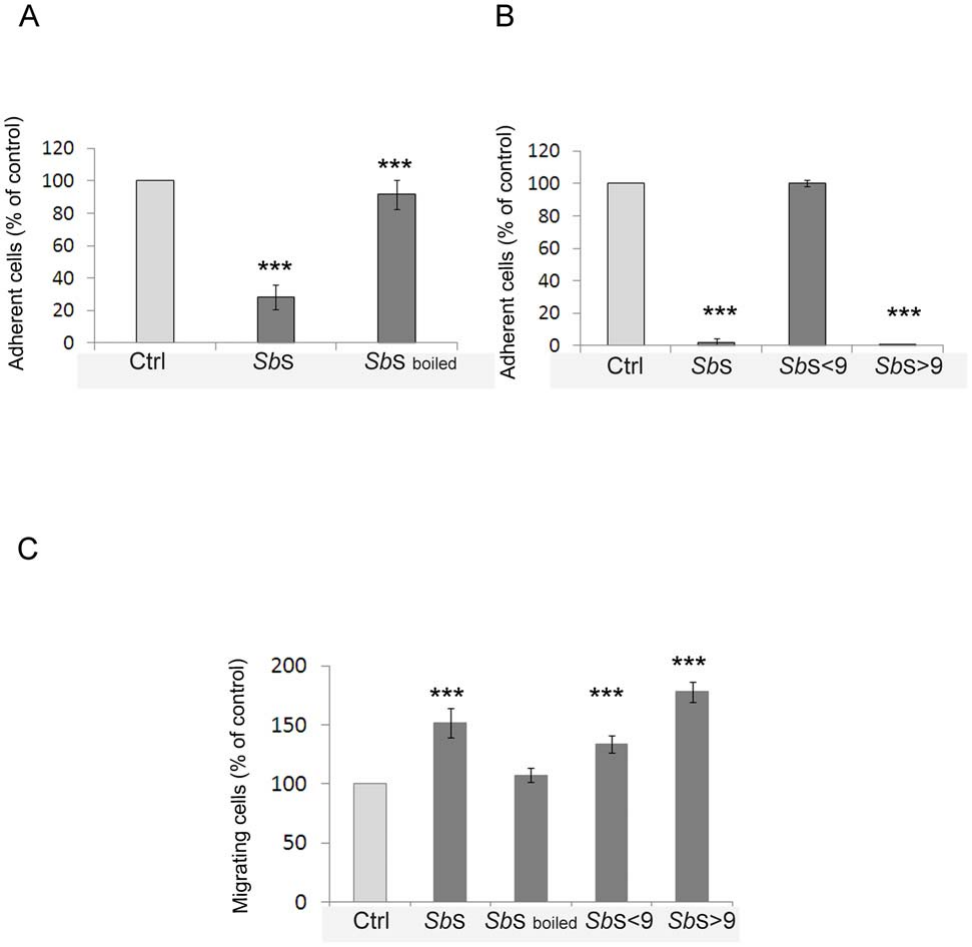
*Sb* secretes several molecules that differentially regulate both cell adhesion and migration. (A) Isolated HCT-8/E11 cells were treated with or without *Sb*S (*Sb*S), boiled *Sb*S (*Sb*S boiled) and plated on 3.15 µg/ml vitronectin. Cell-vitronectin adhesion was evaluated as described in Methods. Results are expressed as the percentage of cell adhesion. Data represent the mean+SD of 3 separate experiments. (B) Isolated HCT-8/E11 cells were treated with or without *Sb*S (*Sb*S), or fractionated *Sb*S (>9 kDa or <9 kDa) and plated on 3.15 µg/ml vitronectin. Cell-vitronectin adhesion was evaluated as described in Methods. Results are expressed as the percentage of cell adhesion. Data represent the mean+SD of 3 separate experiments. (C) HCT-8/E11 cell monolayers were wounded and incubated without (Ctrl) or with *Sb*S (*Sb*S), boiled *Sb*S (*Sb*S *boiled*) or fractionated *Sb* supernatant (>9 kDa or <9 kDa) for 5 h. Wound closure was determined as described in Methods. Results are expressed as the percentage of cell migration compared to control. Data represent the mean+SD of 2 separate experiments. *** P<0.001.

We next sought to better characterize the active component(s) in *Sb*S that blocked cell adhesion to Vn and induced cell migration. We first determined the heat stability of the active component. As illustrated in figure **9A**, boiling the *Sb*S for 10 min abrogated the inhibitory effect of *Sb*S on cell-Vn adhesion. Similar results as those obtained for cell adhesion were observed for cell migration (Figure **9C**), indicating that the blockade of both cell adhesion to Vn and induction of cell migration involved one or more proteins. We next examined the activity of *Sb*S after passage through a 9 kDa cut-off filter. As illustrated in Figure **9B** we found that the filtrate did not retain *Sb*S activity in cell adhesion to Vn, clearly indicating that the active component(s) has a molecular weight higher than 9 kDa. Interestingly, although the filtrated fraction contained motogenic molecules, the flow-through filtration (<9 kDa) fraction was also able to increase cell migration. This indicates that *Sb*S contains at least two molecules capable to modulate cell migration (Figure **9C**).

## Discussion

Intestinal epithelial restitution, proliferation and differentiation are all requirements for wound healing, a process disrupted in infectious or inflammatory bowel diseases, such as ulcerative colitis and Crohn's disease [32]. Therefore, complete remission of such diseases requires both the cessation of inflammation and the repair of damaged epithelium. The development of novel therapies that accelerate the repair of intestinal epithelium has recently begun and various molecules are now being considered for clinical use. These include epidermal growth factor in combination with mesalamine [33], keratinocyte growth factor [34], and hepatocyte growth factor [35]. However, a better understanding of the biological effects of these molecules must be ascertained, not least to identify any undesired secondary effects, such as tumorigenesis. Clinical trials have suggested that *Sb* can be effective in the treatment of gastroenteritis and IBD [18] through the modulation of host cell signaling pathways implicated in the pro-inflammatory response. Moreover, we previously demonstrated both *in vitro* and in mice that *Sb* promoted intestinal restitution [15].

In the present study, we analyzed the effect of *Sb* on the capacity of intestinal epithelial cells to heal a wound. According to our findings, the impact of *Sb* on epithelial cells can be summarized as follows: (1) *Sb*S contains heat-sensitive compounds that decreased αvβ5 integrin-mediated adhesion to Vn by competing with the integrin; (2) *SbS*-mediated changes in cell adhesion to Vn results in a reduction of the αvβ5 signaling pathway; (3) This perturbation is related to *Sb*-induced cell migration. According to these data, we postulated that *Sb*S contains motogenic factor(s) that improved intestinal cell restitution by down-modulating the αvβ5 integrin-Vn interaction.

We demonstrated that *Sb*S contains factors that blocked the cell-Vn interaction. Indeed, our adhesion assays showed that *Sb*S competed with αv integrin subunit in binding to Vn. Indeed, (1) *Sb*S blocked cell adhesion to Vn and (2) addition of *Sb*S onto Vn prior to cell plating impaired cell adhesion. This blockade is selective since *Sb*S did not block the cell interaction with Fn (this paper), laminin or type I collagen [15]. Moreover this phenomenon could be common to all intestinal epithelial cell lines since the same results were observed using HT29-D4 cells, another intestinal cell line. Vn associates with a wide variety of ligands including αv integrin, IGF-I family members, and urokinase plasminogen activator receptor [36] [11], [37]. The binding site(s) present in Vn for *Sb*S factors remain to be elucidated. One might postulate that the tripeptide sequence RGD, involved in binding integrin to both Fn and Vn, does not play a role in this process since *Sb*S did not block cell interaction with Fn. Further studies are required to explore whether sequences flanking the RGD peptide could interact with *Sb*S , as they have been reported to be important for integrin selectivity [38]. On the other hand, other Vn domains could be involved in the interaction with compound(s) found in *Sb*S. For example, Protein E from *Hemophilus influenza* competed with αv subunit containing integrins for binding to Vn by interacting with domains located at the heparin binding regions of Vn [39]. Moreover, the high molecular weight form of kinninogen confers a strong anti-adhesive function upon integrin-mediated cell interaction with Vn [40]. Nevertheless, we reported in this study that the anti-adhesive factor(s) found in *Sb*S is a (are) heat-sensitive compound (s) with a molecular weight higher than 9 kDa.

It is now clear that Vn plays a crucial role in many biological processes including cell migration, adhesion, tissue repair and angiogenesis [41]. Interestingly, other studies have suggested potential roles of Vn in microbial colonization and serum resistance. According to this, recent findings unveiled that many bacterial species, including *C. difficile* and *H. Pylori* interact with Vn [32]. The functionality of these interactions in pathogenesis has not been fully elucidated, although Vn most likely functions as a bridge between bacteria and epithelial cells [32]. Therefore we could postulate that *Sb*S contains compounds that block Vn-enterocyte interactions and then inhibits adhesion of pathogens to host cells. Further work is needed to explore this putative antimicrobial activity in more depth.

Activation of αvβ5 integrin leads to remodeling of the actin cytoskeleton through the formation and activation of signaling complexes called “adhesion complexes”. These multi-molecular complexes contain enzymes and scaffolding molecules including FAK and paxillin [42]. Activation of these signaling molecules, following αv integrin activation leads to the modulation of cell migration [42]. Several arguments suggest that *Sb*S promotes dynamic changes in the αvβ5 integrin affinity for its ligand. Firstly, immunolocalization of both αv integrin subunit and paxillin showed that *Sb*S decreased the area of focal adherence structures. Secondly, this *Sb*S-induced reorganization of focal adherence structures was associated with both a decrease in FAK tyrosine phosphorylation on the Y397 residue upon cell adhesion to Vn and an increase in αvβ5 dependent migration. FAK is a key mediator of intracellular signaling by integrins and may serve as conduits for the transmission of the force necessary for cell migration and bidirectional signaling between the cell interior and its environment [43]. FAK activation leads to the stimulation of other signaling proteins such as paxillin, thereby activating various signaling pathways crucial in the regulation of cell adhesion and migration [43]. Moreover, signals that modify tyrosine phosphorylation may influence mucosal wound healing [44]. Therefore, we suggest that *Sb*S regulates the strength of the αvβ5 integrin/Vn interaction and consequently modulates FAK tyrosine phosphorylation, leading to a change in αvβ5 integrin-dependent migration.

Changes in cell-ECM interactions occur spatially and temporally during intestinal wound healing. Several *in vivo* and *in vitro* studies in the gut indicate the requirement of the binding and interaction of ECM-specific integrins for this restitution, such as α3β1, α6β1, α6β4 laminin-binding integrins, α2β1 collagen-binding integrin or αv-subunit Fn-binding integrin [7], [45]. We recently provided data indicating that *Sb* exerts at least some of its motogenic effect through the activation of the collagen receptor α2β1 integrin [15]. Here we have reported that *Sb*S also exerts some of its motogenic effect via down-modulation of the interaction between αvβ5 integrin and Vn. Indeed, (1) inhibition assays using anti-integrin mAbs demonstrated that αvβ5 integrins participate in the *Sb*S-induced cell migration. Given the ligand-binding properties of these integrins, it appears likely that Vn supports this process. (2) Both cell adhesion and cell spreading to Vn, two processes required for cell migration, were altered by *Sb*S. (3) *Sb* promoted the reorganization of αvβ5 integrins into adhesive structures localized at the leading edge of the cells. These are crucial to migration and are themselves regulated by *Sb*S. Moreover, *Sb*S was shown to promote the redistribution of the Vn receptor in the mouse intestine. This redistribution was observed for both epithelial and non-epithelial cells. In line with these data, some studies have indicated a crucial role of Vn in regulating cell migration during wound healing [46], [47].

Cell migration requires the dynamic interaction between cells and the substratum on which they are attached and over which they migrate [48]. Changes in the density of ligand, integrin repertoire, ligand-binding affinity and/or cytoskeletal associations are all key determinants of cell migration speed [48], [49]. In the present study, we showed, by flow cytometry analysis, that *Sb*S did not alter the concentration of αvβ5integrin or, in previous work, α2β1integrin [15]. However, our data strongly suggest that *Sb*S dynamically regulates the assembly and disassembly of adhesions that are essential for optimum cell migration. Indeed, to promote cell restitution, *Sb*S activates the α2β1 integrin collagen receptor [15] whereas it down-regulates the αv integrin interactions with Vn (this work).

*Sb* acts as a shuttle that could liberate, during the intestinal transit, at least 1500 molecules that have not been totally characterized [50]. This large number of secreted peptidic and non-peptidic factors, including proteases, phosphatases and polyamines, may at least partially explain why *Sb* has pleiotropic effects on intestinal mucosa and also has therapeutic effects on such a wide variety of gastrointestinal disorders [14], [31], [51]. Some of the molecules, as yet unidentified, can interfere with host cell signaling pathways and are therefore able to modulate host cell behavior including intestinal mucosal inflammatory, secretory, and barrier functions [50]. Moreover, *Sb* produces other factors that reduce inflammation by blocking NF-κB and MAPK activation [26], [52] and enhancing PPAR-γ expression [53]. On the other hand, conditioned medium of *Sb* was shown both *in vitro* and *in vivo* to modulate host signaling pathways involved in the regulation of cell motility including the MAPK and FAK pathways [15], [26]. Although the motogenic molecule(s) secreted by *Sb* remain to be elucidated we have reported here that *Sb*S contains two groups of heat-sensitive compounds capable to increase intestinal restitution: one group with a molecular weight lower than 9 kDa and another with a molecular weight higher than 9 kDa. We can postulate that this latest class of molecules corresponds to the same as those involved in the down-modulation of cell adhesion to Vn.

In conclusion, this report demonstrates that *Sb*S contains various heat-sensitive motogenic factors that can improve intestinal restitution. These factors exerted their effect through multiple pathways, including the dynamic fine regulation of integrin-mediated adhesion to the ECM. This could be of major importance in diseases characterized by severe mucosal injury, as seen in IBD or infectious gastroenteritis.

## Supporting Information

Video S1 **HCT-8/E11 cell monolayers were wounded as described in** **Methods** **, incubated with** ***Sb*** **supernatant (dilution 1/8), then placed in a temperature and CO2-controlled chamber mounted on a Nikon TE2000 inverted microscope.** Images were captured every 5 minutes for a total observation period of 5 h, using a Cool SnapHQ camera (Princeton Instrument) through a 10× objective lens.
(MOV)

Video S2 **HCT-8/E11 cell monolayers were wounded as described in** **Methods** **and incubated with** ***Sb*** **S.** Cell monolayers were further incubated with or without function-blocking anti- αv integrin mAb 1 h before wounding and during cell migration. Plates were placed in a temperature and CO2-controlled chamber mounted on a Nikon TE2000 inverted microscope. Images were captured every 5 minutes for a total observation time of 5 h using a Cool SnapHQ camera (Princeton Instrument) through a 10× objective lens.
(MOV)
